# Is intensity the most important factor in determining the amount of prior work accumulated that affects cyclists’ acute durability? A systematic review

**DOI:** 10.1007/s00421-025-05885-0

**Published:** 2025-07-04

**Authors:** Jose Luis Sánchez-Jiménez, Jose-Antonio Salas-Montoro, Manuel Mateo-March, Jose Ignacio Priego-Quesada, Mikel Zabala, Juan-José Pérez-Díaz

**Affiliations:** 1 Research Group in Sports Biomechanics (GIBD), Department of Physical Education and Sports, University of Valencia, 46010 Valencia, Spain; 2 Department of Physical Education and Sport, Faculty of Sport Sciences, University of Granada, Cam. de Alfacar, 21, 18071 Granada, Spain; 3 Sports Research Centre, Department of Sports Sciences, Miguel Hernández University, Elche, Spain

**Keywords:** Fatigue resistance, Cycling performance, Mean maximal power

## Abstract

**Purpose:**

This study aimed to determine how exercise intensity influences the amount of work required to induce changes in cyclists’ acute durability and to evaluate the suitability of using kilojoules (kJ) as a metric for fatigue monitoring.

**Methods:**

A systematic review was conducted following PRISMA guidelines. Web of Science, Medline, and Scopus were searched for studies assessing the relationship or effect between prior accumulated work and performance reductions in cyclists. Inclusion criteria required studies to measure power output after fatigue induced within a single session, with prior work quantified in kJ or other training load metrics.

**Results:**

Twenty-one studies were included in the systematic review. The primary finding was that high-intensity efforts (e.g., above critical power) led to greater power output reductions with lower accumulated work compared to low-to-moderate intensity efforts. Across studies, power output declines of 10–20% were observed after 2.5–15 kJ kg⁻^1^ of prior high-intensity work, whereas similar or greater work volumes at lower intensities resulted in smaller performance decrements. While kJ was the most commonly used fatigue metric, it does not account for intensity, limiting its accuracy in durability assessments.

**Conclusions:**

Exercise intensity plays a crucial role in determining durability-related performance declines. The exclusive use of kJ as a fatigue metric may be insufficient, and alternative approaches incorporating intensity are needed. These findings have implications for training prescription and race strategies, emphasizing the need for intensity-specific workload quantification.

**Registration:**

OSF project no.: osf.io/kcg53.

## Introduction

Situations involving decisive movements for victory in professional cycling races often occur in the event’s final stages, when cyclists are under fatigued conditions (Erp et al. 2021). For this reason, the impact of fatigue on performance, traditionally referred to as “fatigue resistance”, is commonly studied in cycling (Hawley et al. 1997; Morris et al. 2008). However, in recent years more emphasis has been placed on the term “durability” (Muriel et al. 2022; Valenzuela et al. 2023; Spragg et al. 2023a), which can be defined as “the time of onset and the magnitude of any deterioration in physiological profiling characteristics over time during prolonged exercise” (Maunder et al. 2021). Unlike fatigue resistance, which focuses on the ability to maintain performance despite fatigue, durability emphasizes the onset and magnitude of performance deterioration over time during prolonged exercise, often assessed by measuring performance after different accumulated work quantities (Erp et al. 2021; Leo et al. 2021). Although this concept has gained traction, the literature lacks a clear synthesis of how triggering factors, such as exercise intensity, specifically affect durability.

The use of mechanical work measured in kilojoules (kJ) to quantify fatigue has facilitated the establishment of relationships between fatigue and performance. However, while kJ is widely used to quantify fatigue, its inability to account for exercise intensity limits its accuracy in predicting durability decline, as recent studies have demonstrated (Mateo-March et al. 2024; Spragg et al. 2024). While the dose of high-intensity exercise related to power output (PO) reduction varies between studies, with doses ranging from 7.5 to 15 kJ kg^–1^ (Mateo-March et al. 2024; Barranco-Gil et al. 2024), these discrepancies suggest that additional factors, such as intensity distribution, should be considered when assessing fatigue-related declines in performance. To quantify training load and its effects on fatigue, various metrics have been developed using physiological and perceptual data outcomes. Training Impulse (TRIMP), session Rating of Perceived Exertion (sRPE), and Training Stress Score (TSS) are commonly used to estimate the stress induced by training or competition, derived from heart rate, subjective effort perception, and PO, respectively (Erp et al. 2019a, b). Mechanical work, measured in kJ, has also been proposed as an alternative method for quantifying load (Erp et al. 2021; Leo et al. 2022). Nonetheless, the literature reveals a critical limitation: the same amount of work performed at high intensity (above critical power [CP]) or low to moderate intensity (below CP) does not produce the same effect on performance (Mateo-March et al. 2024; Spragg et al. 2024), indicating that work alone may not adequately reflect accumulated fatigue. This review addresses this gap by systematically evaluating how intensity, beyond work volume, establishes durability in cycling.

The aim of this systematic review was to determine how exercise intensity influences the amount of work necessary to induce changes in cyclists’ acute durability, along with assessing the suitability of using kJ for fatigue monitoring. While prior research has primarily quantified fatigue through work volume, this review offers a novel approach by exploring the interplay between intensity and durability across a wide range of experimental and competitive protocols. We hypothesized that intensity would be the most determining factor in PO reduction during prolonged periods of cycling, and that kJ alone will not adequately predict the performance reduction in cyclists.

## Methods

### Search methodology

The systematic review was conducted in accordance with the Preferred Reporting Items for Systematic Reviews and Meta-Analyses (PRISMA) guidelines (Page et al. 2021). The PICO question established was as follows: Which amount of fatigue impacts cyclists’ performance assessed through power output reduction? Is mechanical work the most effective variable to assess and monitor fatigue? Three databases were consulted, Web of Science, Medline (via PubMed), and Scopus, on September 18th, 2024, using specific search strings tailored to each database. For Web of Science, the query was TS = ((“durability” OR “fatigue”) AND (“cycling” OR “cyclist”) AND (“load” OR “work” OR “workload”) AND “power output”); for Medline, ((“durability” OR “fatigue”) AND (“cycling” OR “cyclist”) AND (“load” OR “work” OR “workload”) AND “power output”) with filters for English and Spanish; and for Scopus, TITLE-ABS-KEY((“durability” OR “fatigue”) AND (“cycling” OR “cyclist”) AND (“load” OR “work” OR “workload”) AND “power output”)). Each database employed its own term mapping, meaning search terms were adapted to match database-specific indexing (e.g., MeSH terms in PubMed included “Bicycling” and “Fatigue,” while Web of Science used topic searches). All articles retrieved from the databases were exported to Zotero (version 7.0, Corporation for Digital Scholarship, Vienna, USA) to remove duplicates. The systematic review was registered in the Open Science Framework (OSF): https://osf.io/kcg53.

Subsequently, the first screening process was carried out by reviewing the titles and abstracts of the articles, followed by the eligibility process, which involved full-text reading of the selected articles. Only studies that met all predefined criteria were considered for inclusion.

### Inclusion and exclusion criteria

The review included studies that were published in English or Spanish and focused on the impact of fatigue on cyclists’ performance. Studies were excluded if they were books, book chapters, reviews, conference papers, or involved participants with chronic diseases (e.g., diabetes, cardiovascular conditions…) or acute injuries requiring medical intervention. During the eligibility assessment, the following criteria were considered: the use of PO measurements, evaluation of cycling performance in both non-fatigued (fresh) and a fatigued state after a fatigue-inducing cycling or ergometer session, fatigue induced within the same session either on a bike or an ergometer, and the quantification of prior fatigue (e.g., kJ, TSS, TRIMP, or sRPE) or the ability to calculate these metrics from the study data. The criterion requiring fatigue to be induced within the same session was chosen to ensure consistency in assessing acute fatigue effects, though this may exclude valuable multi-day studies (e.g., Grand Tour simulations); this limitation is acknowledged and justified by the focus on acute durability responses rather than chronic fatigue accumulation.

### Study selection and data extraction

The initial screening was conducted by reviewing the titles and abstracts. Afterward, the full text of the selected articles was assessed for eligibility. Once the final list of articles included in the review was established, the following data were extracted from each article: (1) sample size and participants’ level, (2) performance indicators, (3) fatigue protocol, (4) method of fatigue measurement, and (5) main results (specifically, the magnitude of PO reduction and, where reported, its statistical significance). All tasks were conducted in parallel by two authors, and in cases of disagreement, a third author was consulted to reach a consensus.

### Bias assessment

The quality of the observational studies included in the systematic review was assessed using the Newcastle–Ottawa Scale (NOS) (Wells et al. 2000), a tool that evaluates selection, comparability, and outcome quality in non-randomized studies. The bias of quasi-experimental studies was assessed using the ROBINS-I Scale (Sterne et al. 2016), which assesses the risk of bias in non-randomized intervention studies across domains such as confounding and selection. Lastly, studies with randomized conditions were analysed using the Cochrane Risk of Bias Tool for Randomized Trials (RoB 2) (Sterne et al. 2019), a framework for assessing bias in randomized trials, including randomization process and outcome measurement. Two authors worked independently on the assessment, and a third author was consulted to resolve disputes through discussion until consensus was reached, guided by predefined criteria from each tool’s guidelines.

## Results

### Study selection

**Fig. 1:**
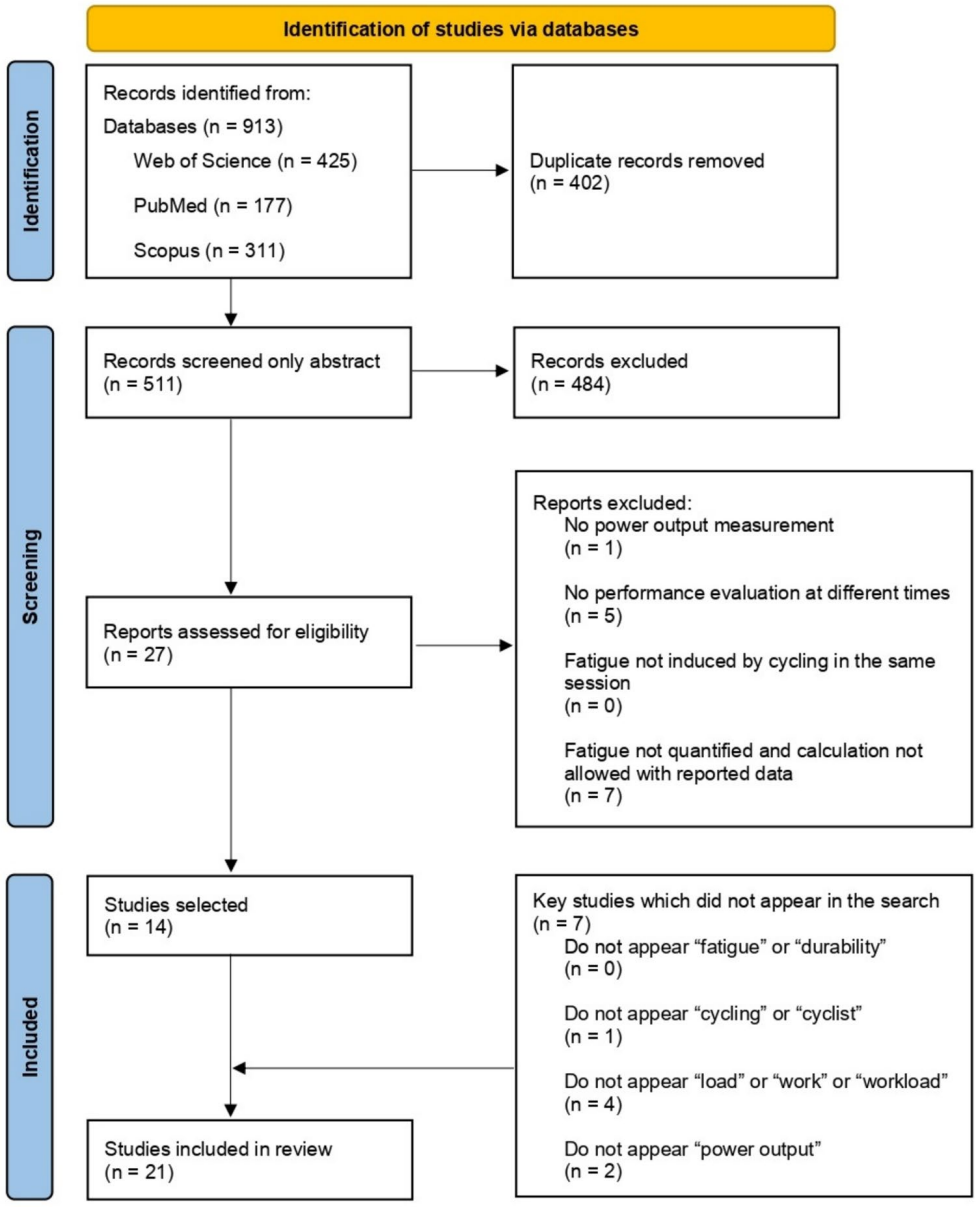
Flow diagram of the process followed for study selection

Of 913 initial studies retrieved from Web of Science (n = 425), Medline (n = 177), and Scopus (n = 311), after removing duplicates, 511 unique studies remained. Screening of titles and abstracts excluded 484 articles, leaving 27 for full-text review, of which 14 met inclusion criteria. An additional 7 studies were identified through other sources, such as reference lists and expert recommendations (Fig. 1).

### Bias assessment

**Fig. 2:**
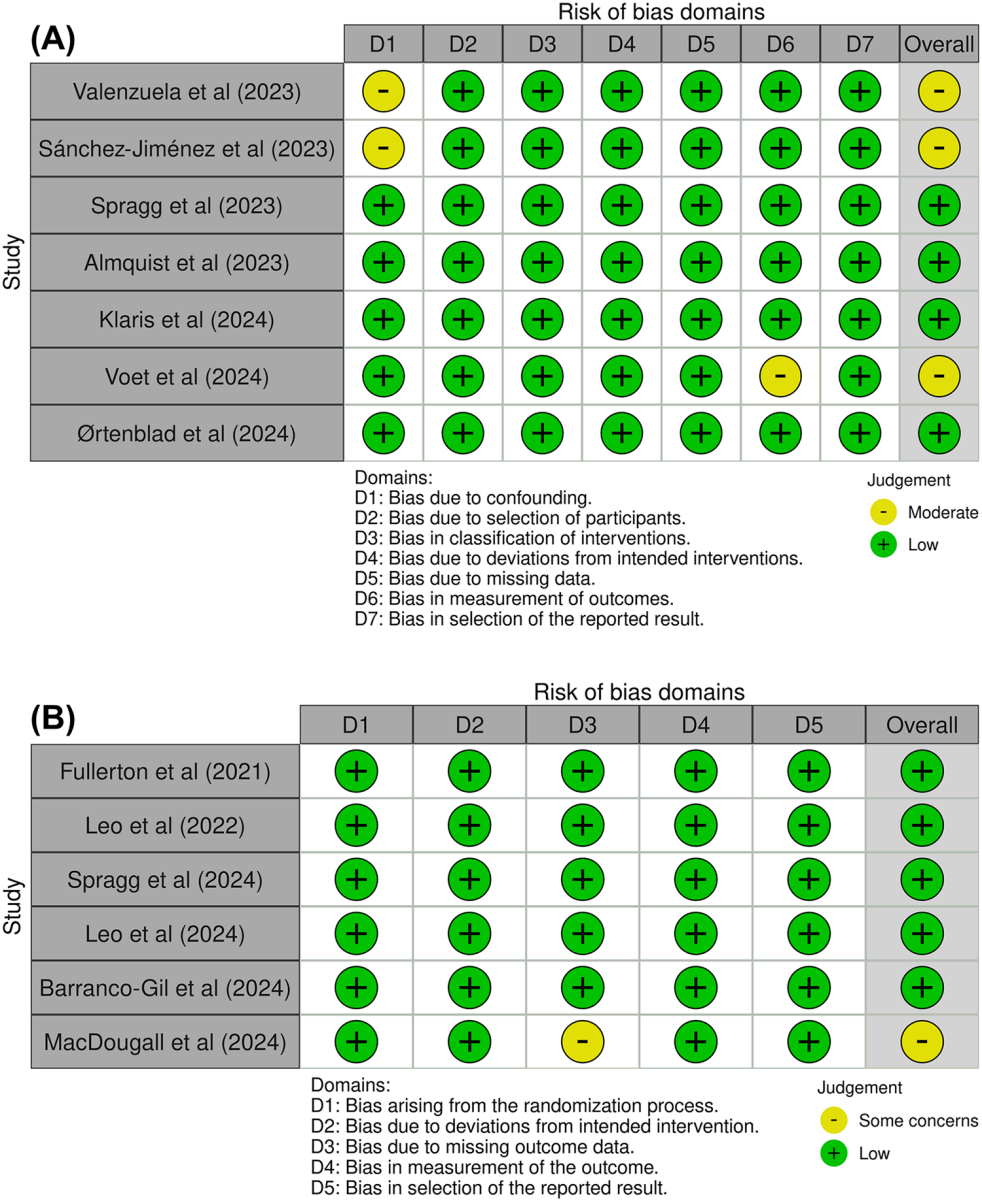
The risk of bias for each study. **A** ROBINS-I and **B** RoB-2. Created with “robvis” application (McGuinness and Higgins 2021)

The results of the bias assessment are presented in Fig. 2. The number of articles assessed with NOS, ROBINS-I, and RoB-2 scales were eight, seven, and six, respectively. For the NOS scale, three studies obtained seven points overall, and five obtained eight points. The non-exposed selection and the comparability of groups were the items with the lowest reported values. In the remaining items, all studies achieved maximum scores. For ROBINS-I, 71.4% of studies had a low overall risk, with moderate risk primarily in Bias due to confounding (28.6%) and Bias due to missing data (14.3%); other domains showed a low risk across all studies. For RoB-2, 83.3% of studies had low overall risk, with Bias due to missing outcome data raising concerns in 16.7%; the remaining domains showed no significant issues in most studies.

### Sample characteristics

Table 1 shows the results extracted from the articles included in the systematic review. Data extracted from the figures were obtained using the plot digitalized application (Drevon et al. 2017). A total of 585 participants were included in the studies analysed in this review. Of these, 384 were categorized according to competitive cycling levels: junior (27 males) (Barranco-Gil et al. 2024; Gallo et al. 2022), Under 23 (U23) (11 males) (Leo et al. 2024), U23 Continental Team (70 males) (Spragg et al. 2023a, b; Leo et al. 2021; Gallo et al. 2022), Continental (23 males) (Ørtenblad et al. 2024; Voet et al. 2024), Pro Team (PT) (87 males) (Muriel et al. 2022; Valenzuela et al. 2023; Leo et al. 2021, 2024; Mateo-March et al. 2022a), and WorldTour (WT) (132 males) (Muriel et al. 2022; Mateo-March et al. 2022a, 2024; Gallo et al. 2022; Leo et al. 2024). An additional group of 34 males was reported as belonging to WT and PT categories, but their exact distribution was not specified (Erp et al. 2021; Leo et al. 2021). Moreover, 14 male participants were described as professional cyclists without specifying their team category (Erp et al. 2022).

**Table 1:** Results extracted from the studies included in the review

Study (year)	Sample	Performance Assessment	Fatigue Protocol	Fatigue Measurement	Results: Performance Decline
Fullerton et al. (2021)	N = 6 (M); 5 (F) Active and well-trained	Time-to-task failure	5 × 30-min constant PO Different intensities below and above MLSS 15-min constant PO at MLSS 45-min constant PO at MLSS	**kJ**	Prior exercise above MLSS causes a greater performance decline than exercise below MLSS
Leo et al. (2021)	N = 8 (M) WT and PT N = 9 (M) U23 Continental	PP 5s, 10s, 15s, 30s. 1-min, 2-min, 5-min, 12-min, 20-min, 30-min	Data collected during the five stages of Tour the Alps (2018–2019) Characteristics of the editions: 714 ± 10 km 13,435 ± 199 m of climbing	**kJ** 1000 kJ 1500 kJ 2000 kJ 2500 kJ 3000 kJ	After 3000 kJ **Professional** 5s: -1.71 W kg^–1^ 1-min: -1.06 W kg^–1^ 5-min: -0.87 W kg^–1^ 20-min: -0.81 W kg^–1^ **U23** 5s: -3.21 W kg^–1^ 1-min: -1.98 W kg^–1^ 5-min: -1.26 W kg^–1^ 20-min: -0.91 W kg^–1^ *Extracted from figures
Van Erp et al. (2021)	N = 26 (M) WT and PT	PP 10s, 1-min, 5-min, 20-min	Database from 2012 to 2019 85 seasons 207 ± 35 files per season 75.3% from training 22% from races 2.7 from TT	**kJ** 0 kJ kg^–1^ 10 kJ kg^–1^ 20 kJ kg^–1^ 30 kJ kg^–1^ 40 kJ kg^–1^ 50 kJ kg^–1^	After 50 kJ kg^–1^ **Climbers** 10s: -1.48 W kg^–1^ 1-min: -1.03 W kg^–1^ 5-min: -0.39 W kg^–1^ 20-min: -0.45 W kg^–1^ **Sprinters** 10s: -2.45 W kg^–1^ 1-min: -1.25 W kg^–1^ 5-min: -0.55 W kg^–1^ 20-min: -0.78 W kg^–1^ *Difference between moments calculated from the data reported
Gallo et al. (Gallo et al. 2022)	N = 15 (M) Junior N = 21 (M) U23 Continental N = 17 (M) WT	PP 10s, 1-min, 5-min, 20-min	One season between 2016 and 2019 was analysed for each cyclist. Training and races were collected	**kJ** 10 kJ kg^–1^ 20 kJ kg^–1^ 30 kJ kg^–1^ 40 kJ kg^–1^ 50 kJ kg^–1^	After 40 kJ kg^–1^ (Junior) and 50 kJ kg^–1^ (U23 and WT) **Junior** 10s: -2.77 W kg^–1^ 1-min: -3.08 W kg^–1^ 5-min: -1.85 W kg^–1^ 20-min: -2.0 W kg^–1^ **U23** 10s: -2.76 W kg^–1^ 1-min: -2.11 W kg^–1^ 5-min: -0.96 W kg^–1^ 20-min: -0.98 W kg^–1^ **WT** 10s: -0.94 W kg^–1^ 1-min: -0.56 W kg^–1^ 5-min: -0.1 W kg^–1^ 20-min: -0.20 W kg^–1^ *Difference between moments calculated from the data extracted from the figures
Mateo-March et al. (2022a)	N = 66 (M) WT N = 46 (M) PT	PP 10s, 1-min, 5-min, 20-min, 60-min, 120-min	Seasons between 2013 and 2021 855 seasons were collected, corresponding 8 ± 5 per cyclist 103,102 files ~ 80% of the files were training sessions and ~ 20% races	**kJ** 0 kJ kg^–1^ 15 kJ kg^–1^ 25 kJ kg^–1^ 35 kJ kg^–1^ 45 kJ kg^–1^	The magnitude of performance decline increased proportionally with accumulated work (e.g., a − 1.6% to − 3.0% decline after 15 kJ kg⁻^1^ and a − 6.0% to − 9.7% decline after 45 kJ kg⁻^1^), with greater declines in PT compared to WT cyclists’
Van Erp and Lamberts (2022)	N = 14 (M) Professional	PP 5s, 10s, 15s, 30s, 1-min, 3-min, 5-min, 10-min, 20-min, 60-min	Seasons between 2013 and 2019 43 seasons were collected corresponding 3 ± 1 per cyclist 1324 files from races	**kJ** MMP relative to the workload of the race	Work at MMP occurred and percentage of total work done **TOP5** 5s: 28 (82%) W kg^–1^ 1-min: 26 (77%) W kg^–1^ 5-min: 22 (65%) W kg^–1^ 20-min: 27 (70%) W kg^–1^ **NON-TOP5** 5s: 17 (50%) W kg^–1^ 1-min: 17 (51%) W kg^–1^ 5-min: 18 (52%) W kg^–1^ 20-min: 24 (59%) W kg^–1^ *Extracted from figures
Leo et al. (2022)	N = 9 (M) PT	TT 12-min	Two training camps within one month of each other. Order of MIC and HII randomized 12-min fresh (1) 150-min moderate continuous work (MIC; < 70% peak HR) (2) 150-min race simulation with random high intensity efforts (HII; > 80% peak HR) 12-min fatigue	**kJ** 237 kJ of difference between methods MIC: ~ 1750 kJ HIIT: ~ 2000 kJ *Estimation	After High Intensity: 12-min: -0.46 W kg^–1^ Difference of 0.5 W kg^–1^ in 12-min between protocols in fatigued state
Muriel et al. (Muriel et al. 2022)	N = 7 (M) PT N = 8 (M) WT	PP 5s, 10s, 30s, 1-min, 5-min, 10-min, 20-min, 30-min	Race data from La Vuelta 2020 2895 km 45,991 m elevation	**kJ** 0 kJ kg^–1^ 15 kJ kg^–1^ 25 kJ kg^–1^ 35 kJ kg^–1^	After 35 kJ kg–1 **PT** 5s: -1.02 W kg^–1^ 1-min: -0.85 W kg^–1^ 5-min: -0.39 W kg^–1^ 20-min: -0.16 W kg^–1^ **WT** 5s: -0.66 W kg^–1^ 1-min: -0.33 W kg^–1^ 5-min: -0.32 W kg^–1^ 20-min: -0.01 W kg^–1^ *Difference calculated based on the data extracted from figures
Valenzuela et al. (2023)	N = 12 (M) PT	TT 20-min	**Session 1** Graded exercise test **Session 2** **TT:** 20-min 48h in between **Session 3** **FP:** Outdoor ride until accumulating ~ 40 kJ kg^–1^ without exceeding functional threshold power **TT:** 20-min	**kJ** 39.8 ± 1.2 kJ kg^–1^	20-min: -11 ± 12 W
Spragg et al. (Spragg et al. 2023b)	N = 10 (M) U23 Continental	CP 3-min 12-min	**Laboratory Testing** Randomized related to CP tests Graded exercise test 6-min at 200W 6-min at 300W **Session 1** **TT:** 3-min, 12-min **Session 2** **FP:** 20-min at 50–70% of CP 5 × 8-min 105–110% of CP **TT:** 3-min, 12-min	Not available	CP: -0.15 W kg^–1^ W’: -4.64 kJ *Extracted from the figures
Sanchez-Jimenez et al. (2023)	N = 10 Trained/Developmental (2022)	PP and CP 1-min, 5-min, 20-min	**Session 1** **PP:** 1-min, 5-min, 20-min **Session 2** **FP:** 10-min (95% of MMP20) + 1-min(maximal) *Until PO decay ≥ 20% **PPF:** 1-min, 5-min, 20-min	**kJ** 237.3 ± 18.5 kJ 3.5 ± 0.2 kJ kg^–1^	1-min: -0.8 (9%) W kg^–1^ 5-min: -0.3 (5.9%) W kg^–1^ 20-min: -0.2 (4.1%) W kg^–1^
Spragg et al. (2023a)	N = 30 U23 Continental	PP from MMP 2-min, 5-min and 12-min	**Session 1** Graded exercise test **Session 1** **TT:** 2-min, 5-min **Session 2** **TT:** 12-min *Consecutive days Two PP (fresh and fatigued) in each period were created	**kJ** 2000 kJ	CP: -0.30 W kg^–1^ W´: -3.02 kJ 2-min: -0.68 W kg^–1^ 5-min: -0.44 W kg^–1^ 12-min: -0.37 W kg^–1^ *CP and W´ were estimated from the efforts. MMP were extracted from figures
Almquist et al. (Almquist et al. 2023)	N = 100 (M) N = 19 (F) Junior, U23 and Senior cyclists’ of the Norwegian national team	**TT** 5-min	Physiological testing from 2015 to 2021. Three consecutive days **Session 1** BLa profile test Incremental test **TT:** 40-min submaximal, 5-min **Session 2** 6-s (2x), 15-s, 30-s, 60-s and 12-min **Session 4** **TT:** 30-min	**kJ**	Percentage of decrease W_max_ vs 5-min **Females** Junior: 21.1 ± 3.5% U23: 16.5 ± 3.6% Senior: 18.9 ± 2.4% **Males** Junior: 17.2 ± 0.17% U23: 15.9 ± 1.5% Senior: 15.7 ± 3.1%
Klaris et al. (Klaris et al. 2024)	N = 12 (M) National elite cyclists	TT 10s 7-min	**Session 1** Fatmax testing Incremental test **Session 2** 6-h of race simulation with 10-s and 7-min maximal efforts at rest and each 2h	**kJ** 0-2h: 1660 ± 123 kJ 22 ± 1.8 kJ kg^–1^ 0-4h: 3069 ± 245 kJ 40.6 ± 3.5 kJ kg^–1^ 0-6h: 4610 ± 360 kJ 61.0 ± 5.0 kJ kg^–1^	Variation performance in 7-min: 0-2h: -7W 2-4h: -7W 4-6h: -14W Total: -28W
Spragg et al. (2024)	N = 14(M) Elite/International (2022)	PP and CP 1s, 15s, 3-min, 12-min	**Session 1** **PP:** 15s, 3-min, 12-min **Session 2 & 3 (Randomized)** **FP:** Ride ~ 2000 kJ below 70% of CP [Low intensity continuous protocol] **PPF:** 15s, 3-min, 12-min **FP:** Accumulate ~ 2000 kJ of work with 20-min below 70% of CP and 5 × 8-min at 105–110% of CP [High intensity protocol] **PPF:** 15s, 3-min, 12-min	**kJ** Low Intensity 1985 ± 242 kJ High Intensity 1878 ± 340 kJ	**Moderate Intensity** 1s: -0.56 W kg^–1^ 15s: -0.29 W kg^–1^ 3-min: -0.17 W kg^–1^ 12-min: -0.05 W kg^–1^ **High Intensity** 1s: -1.57 W kg^–1^ 15s: -1.47 W kg^–1^ 3-min: -0.17W W kg^–1^ 12-min: 0 W kg^–1^ CP Moderate Intensity: 0.007 W kg^–1^ High Intensity: 0.06 W kg^–1^ *Difference calculated from table of results and body mass. CP extracted from figures
Mateo-March et al. (2024)	N = 17 (M) WT	PP and CP 30-s, 5-min, 10-min, 20-min	One season from January to October 283 ± 27 files per cyclist Training and competitions	**kJ** 0 kJ kg^–1^ 2.5 kJ kg^–1^ 5.0 kJ kg^–1^ 7.5 kJ kg^–1^	**Above CP** CP: -0.99 W kg^–1^ 30s: -2.67 W kg^–1^ 5-min: -1.00 W kg^–1^ 10-min: -1.09 W kg^–1^ 20-min: -1.17 W kg^–1^ **Below CP** CP: -0.07 W kg^–1^ 30s: -0.22 W kg^–1^ 5-min: -0.07 W kg^–1^ 10-min: -0.07 W kg^–1^ 20-min: -0.09 W kg^–1^ *Difference calculated from the data reported in the table of results and body mass
Leo et al. (2024)	N = 11 (M) U23 N = 13 (M) PT N = 24 (M) WT	CP 2-min, 5-min, 12-min PP 5-s, 30-s, 1-min, 5-min, 10-min, 20-min, 30-min	**Session 1 & 2 – CP** (Preseason) 2-min, 5-min, 12-min PP from training and competition data	**kJ** 0 kJ kg^–1^ 2.5 kJ kg^–1^ 5.0 kJ kg^–1^ 7.5 kJ kg^–1^	After 7.5 kJ kg^–1^ **U23** 5s: -4.85 W kg^–1^ 1-min: -2.52 W kg^–1^ 5-min: -1.1 W kg^–1^ 20-min: -0.82 W kg^–1^ **PT** 5s: -3.46 W kg^–1^ 1-min: -1.53 W kg^–1^ 5-min: -0.81 W kg^–1^ 20-min: -1.02 W kg^–1^ **WT** 5s: -2.14 W kg^–1^ 1-min: -1.05 W kg^–1^ 5-min: -0.46 W kg^–1^ 20-min: -0.61 W kg^–1^ *Difference calculated based on the data extracted from figures
Voet et al. (2024)	N = 16 (M) Continental	TT 1-min 10-min	Three visits in a season: December, February and July A session includes: Incremental exercise test Performance test (fresh) Outdoor ride to achieve accumulated work Performance test (fatigue) **FP:** Outdoor ride at 3.2 W kg^–1^ **Performance test** 6-min (55% VO_2max_) 6-min (65% VO_2max_) **TT:** 1-min, 10-min	**kJ** 38.7 ± 3.7 kJ kg^–1^ *Accumulation of ride and fresh performance test	**Preseason** 1-min: -70 ± 35 W 10-min: -10 ± 18 W **Start season** 1-min: -69 ± 52 W 10-min: -10 ± 20 W **In season** 1-min: -37 ± 16 W 10-min: -4 ± 12 W
Barranco-Gil et al. (2024)	N = 12 (M) Junior	CP 2-min, 5-min, 12-min	**Session 1 (CP)** 2-min, 5-min, 12-min **Session 2 & 3 (Randomized)** **FP:** Ride at 65% of CP until reach kJ kg^–1^ **CP:** 2-min, 5-min, 12-min **FP:** 60-min warm up and conduct 3-min intervals (~ 115% CP) until reach 15 kJ kg^–1^ **CP:** 2-min, 5-min, 12-min	**kJ** 15 kJ kg^–1^ **TSS** HIIT: 134 ± 14 MIC: 92 ± 8	After moderate ride: CP: -0.0003 W kg^–1^ After high intensity ride: CP: 0.03 W kg^–1^
Ørtenblad et al. (2024)	N = 7 (M) Continental N = 5 Elite level	6-min TT	**Session 1** Submaximal test (substrate and gross efficiency) 6-min TT **Session 2** 4-h of intermittent cycling (1-min at 120% PO_6-min_ each 30-min and 6-s every 60-min) 6-s and 6-min TT	**Estimated kJ** 3100 kJ 43.35 kJ kg^–1^	6-min: -0.55 W kg^–1^
MacDougall et al. (2024)	N = 8 (M) N = 8 (F) Recreationally active	Time to task failure at 80% PPO	**Session 1** Incremental test **Session 2** Time to task failure fresh **Session 3 & 4 (randomized)** **FP:** Warm-up + 10 × 2-min at 80%PPO (38-min) Time to task failure **FP:** 38-min at 54%PPO Time to task failure	**kJ** HIIT: 293.0 ± 76.0 kJ Constant: 296.5 ± 72.7 kJ **sRPE TRIMP** HIIT: 330 ± 45 Constant: 204 ± 80	Time to task failure Fresh: 548 ± 95s After HIIT: 82 ± 29s (84.5 ± 6.1%) After CWR: 208 ± 96s (61.7 ± 18.2%)

*BLa*, blood lactate; *CP*, critical power; *CWR*, constant work rate; *F*, Female; *FP*, fatigue protocol; *HIIT*, high intensity interval training; *M*, male; *MIC*, moderate intensity continuous work; *MLSS*, maximal lactate steady state; *MMP*, mean maximal power; *PO*, power output; *PP*, power profile; *PPF*, power profile under fatigue condition; *PPO*, peak power output; *PT*, ProTeam; *sRPE*, session rating of perceived exertion; *TRIMP*, training impulse; *TSS*, training stress score; *TT*, time trial; *U23*, under 23; *VO*_*2max*_, maximal oxygen uptake; *W*_*max*_, maximal power output during an incremental exercise test to exhaustion; *WT*, WorldTour; *W’*, work capacity above critical power

Based on the classification by McKay et al. (2022), another 29 participants were categorized as Trained/Developmental (10 males) (Sanchez-Jimenez et al. 2023) and Elite/International (19 males) (Spragg et al. 2024; Ørtenblad et al. 2024).

The review also included 16 recreationally active participants (8 males and 8 females) (MacDougall et al. 2024). Other participants were described as Active and Well-Trained (6 males, 5 females) (Fullerton et al. 2021), National Elite (12 males) (Klaris et al. 2024), and part of a national selection team ranging from junior to senior levels (100 males, 19 females) (Almquist et al. 2023).

### Type of study

Different methodologies were followed in the studies included in this review. Data analysis from databases was employed in eight of the studies, analysing either competition data alone or both race and training data together. The races analysed were the Tour of the Alps (Leo et al. 2021) and La Vuelta (Muriel et al. 2022). The remaining study did not specify the professional competition analysed (Erp et al. 2022). Additionally, four studies analysed race and training data (Erp et al. 2021; Spragg et al. 2023a; Mateo-March et al. 2022a, 2024; Gallo et al. 2022).

Laboratory and field testing were utilized in the studies reviewed. Specifically, four studies focused on laboratory testing (Ørtenblad et al. 2024; Voet et al. 2024; MacDougall et al. 2024; Fullerton et al. 2021; Almquist et al. 2023), six were conducted in the field (Valenzuela et al. 2023; Spragg et al. 2024; Barranco-Gil et al. 2024; Leo et al. 2022, 2024; Sanchez-Jimenez et al. 2023) and two studies combined both laboratory and field conditions (Spragg et al. 2023b; Klaris et al. 2024).

### Performance assessment

Cycling performance was assessed using various methods, including time to task failure, time-trials (TT), record power profiles and CP. Time to task failure was utilized in two studies, both conducted at 80% of peak PO (MacDougall et al. 2024; Fullerton et al. 2021). TT were employed in six studies with varying durations and numbers of efforts. Four studies used a single TT, with durations of 5-min (Almquist et al. 2023), 6-min (Ørtenblad et al. 2024), 12-min (Leo et al. 2022) and 20-min (Valenzuela et al. 2023). Additionally, two studies incorporated two TT efforts to combine short and long durations: Voet et al. (2024) used 1-min and 10-min TT, and Klaris et al. (2024) used 10-s and 7-min TT. Mean Maximal Power (MMP) profiles, the most common metric (12 studies), assessed efforts from 5-s to 120-min, with 1-min, 5-min, and 20-min durations predominant in eight studies (Erp et al. 2021; Muriel et al. 2022; Spragg et al. 2023a, 2024; Leo et al. 2021, 2024; Mateo-March et al. 2022a, 2024; Barranco-Gil et al. 2024; Gallo et al. 2022; Erp et al. 2022; Sanchez-Jimenez et al. 2023). CP was also used to assess performance in seven studies (Spragg et al. 2023a, b, 2024; Mateo-March et al. 2024; Barranco-Gil et al. 2024; Leo et al. 2024; Sanchez-Jimenez et al. 2023).

### Fatigue protocol

Fatigue protocols were categorized as follows: Nine studies used unspecified race/training data for fatigue induction (Erp et al. 2021; Muriel et al. 2022; Spragg et al. 2023a; Leo et al. 2021, 2024; Mateo-March et al. 2022a, 2024; Gallo et al. 2022; Erp et al. 2022). Four studies employed prolonged continuous rides (Valenzuela et al. 2023; Voet et al. 2024; Fullerton et al. 2021; Almquist et al. 2023), four used interval-based rides with efforts from 6-s to 20-min (Spragg et al. 2023b; Ørtenblad et al. 2024; Sanchez-Jimenez et al. 2023; Klaris et al. 2024), and four combined both approaches (Spragg et al. 2024; Barranco-Gil et al. 2024; Leo et al. 2022; MacDougall et al. 2024).

### Fatigue quantification

All articles included in the review used mechanical work (kJ) or, where unavailable, calculated it from power and time to quantify prior fatigue. Additionally, some studies considered other metrics such as TSS, TRIMP or sRPE.

## Discussion

The aim of this study was to investigate how exercise intensity influences the amount of work required to induce changes in cyclists’ durability, as well as to evaluate the suitability of using kJ as a metric for fatigue monitoring. The primary finding of this systematic review is that a lower accumulated workload is needed to impair cycling performance when the work is performed at high intensity (e.g., above CP or Functional Threshold Power [FTP]). Additionally, while kJ was the most commonly used metric to quantify prior fatigue, alternative metrics that incorporate exercise intensity, such as TSS or TRIMP, were utilized in only a limited number of studies.

### Impact of intensity and accumulated work on cycling performance

Endurance performance in cycling has traditionally been assessed through maximal oxygen uptake (VO_2max_), exercise economy, and fractional utilization of VO_2max_ (Jones 2024), linked to ventilator or lactate thresholds. These metrics, measured at exercise onset, are not static and decline with fatigue, reflecting an athlete’s ability to sustain these parameters during prolonged efforts (Jones 2024; Jones and Kirby 2025). In cycling, durability –the capacity to resist performance declines after prolonged exercise– is shaped by intensity, accumulated work, and pacing strategies, with experienced cyclists showing greater resilience (Jones and Kirby 2025). This dynamic interplay underpins cycling performance in this review.

Our study confirms that intensity is the most critical factor in determining performance reduction following prior work. Specifically, high-intensity efforts (e.g., above CP), often prescribed through various interval protocols, result in greater performance impairments with less accumulated work compared to protocols conducted at low to moderate intensities (e.g., below CP). For instance, efforts above CP reduced PO by 10–20% with 2.5–15 kJ kg^–1^ of work, whereas low-to-moderate intensity efforts below CP typically yielded < 5% reductions even at higher volumes (Mateo-March et al. 2024; Spragg et al. 2024; Barranco-Gil et al. 2024). High-intensity protocols based on time (Leo et al. 2022), total work (Spragg et al. 2024), or work normalized to body mass (Barranco-Gil et al. 2024), consistently led to reductions in TT performance. However, the magnitude and duration of these performance impairments appear to depend on the TT duration.

While Leo et al. (2022) reported a decrease in 12-min TT performance following a high-intensity protocol, Barranco-Gil et al. (2024) observed similar reductions in a 2-min TT. Interestingly, Spragg et al. (2024) found that high-intensity work decreased PO in shorter efforts (1-s, 15-s, and 3-min) but did not significantly affect 12-min TT performance. This suggests that high-intensity prior work disproportionately affects short efforts due to rapid glycogen depletion and neuromuscular fatigue (Allen et al. 2008), an effect exacerbated by the specific metabolic responses of fast-twitch muscle fibres (Vanhatalo et al. 2016). Longer efforts may be less affected unless fatigue exceeds a critical threshold, depending on protocol specifics. Notably, despite variations in how protocols were prescribed (e.g., duration, absolute total work, or normalized work), the percentage of CP was consistently used to define work intensity across studies.

Other studies in the review examined the effects of fatigue protocols at varying intensities without direct comparisons between methods (Mateo-March et al. 2024; Leo et al. 2024; Spragg et al. 2023b; Ørtenblad et al. 2024; Sanchez-Jimenez et al. 2023; Klaris et al. 2024). Collectively, these studies demonstrate that fatigue impacts a broad spectrum of exercise durations, though the magnitude of performance reduction varies depending on the duration of the effort and the fitness level of the cyclists.

For shorter efforts, Sanchez-Jimenez et al. (Sanchez-Jimenez et al. 2023) and Mateo-March et al. (2024) observed significant PO reductions in 30-s (− 21.6%) and 1-min efforts (− 9.0%), as well as in longer 20-min efforts (− 4.1 to − 19.1%). Similarly, Klaris et al. (2024) reported performance decrements in both 10-s (− 6.5% following 2h) and 7-min (− 7%) TT following a 6-h field race simulation. These findings suggest that fatigue induced by prior exertion can impair performance across a wide range of durations, though the greatest reductions tend to occur in shorter efforts. In longer efforts, Ørtenblad et al. (2024) reported a 10% reduction in 6-min TT PO and a 6% decrease in peak PO, while Spragg et al. (2023b) observed an 11 W reduction in CP. These results align with the broader trend that fatigue affects both short and longer efforts, though the magnitude of impairment may depend on the specific protocol and the cyclists’ training status. A key finding from Leo et al. (2024) highlights the influence of fitness level on durability. While all cyclists required at least 2.5 kJ kg^–1^ above CP to exhibit a significant decline in MMP, U23 cyclists experienced notable declines in all MMP values for efforts lasting ≥ 1-min after prior exertion exceeding 2.5–5.0 kJ kg^–1^ above CP. In contrast, PT and WT cyclists only showed significant reductions after reaching 5.0–7.5 kJ kg^–1^ above CP. This underscores the importance of training status in determining fatigue resistance and the ability to sustain performance under high workloads.

Due to the critical role of intensity in determining performance reduction, the use of kJ as a metric for durability assessment presents a significant limitation. This is because kJ solely quantifies accumulated work (Work = power [W] × time [s]) without accounting for exercise intensity. For instance, two cyclists may accumulate the same amount of work, but if one performs the work at a higher intensity, the resulting performance impairment may differ substantially. This limitation is evident in most of the studies reviewed, where the use of kJ as a measure of prior fatigue may fail to represent accurately the impact of intensity on fatigue. Alternative metrics that integrate both volume and intensity could provide a more precise approach to fatigue quantification. Future research should explore methods that incorporate both effort duration and intensity for a more comprehensive assessment. One potential approach is normalizing mechanical work by the percentage of CP or FTP during the effort to contextualize the work done relative to intensity. Additionally, assessing mechanical work concerning other variables such as time (kJ min^–1^), distance (kJ km^–1^), or Average Ascent Speed (VAM) (kJ VAM^–1^) could yield new insights. This has important implications for training prescription and competition strategies, as underestimating or overestimating fatigue could lead to suboptimal performance outcomes.

Beyond the intensity and total accumulated work, recent studies have underscored the importance of mechanical factors –particularly the torque-cadence relationship– in determining PO sustainability under fatigued conditions. Evidence suggests that the decline in PO observed with fatigue is primarily driven by reductions in cadence, rather than torque (Sanchez-Jimenez et al. 2023; Leo et al. 2025). In contrast, in a non-fatigued (fresh) state, PO appears to depend more on the ability to generate high torque (Leo et al. 2023). Therefore, future research should incorporate these mechanistic variables when evaluating and prescribing durability-oriented training and testing protocols.

### Cyclist level

The articles included in this review indicate that a cyclist’s level is a key factor in determining durability. Specifically, WT cyclists (the highest professional category) require a greater accumulated workload to experience performance declines compared to PT or U23 cyclists. Leo et al. (2021) reported that U23 cyclists showed significant MMP decrements in efforts ≤ 12-min after 1000 kJ, with longer efforts declining at 1500–2500 kJ. In contrast, professionals only showed reductions in 5- and 12-min MMP after 1000 kJ, with other durations declining at 2000–3000 kJ (Leo et al. 2021). Similarly, Gallo et al. (2022) observed lower fatigue resistance in junior cyclists compared to U23 and professionals, attributing this to the shorter race durations in junior categories. WT cyclists’ greater durability likely stems from higher training volumes, superior aerobic capacity, and years of competitive experience, as evidenced by their ability to sustain PO under fatigue (Leo et al. 2021; Gallo et al. 2022). They also found fatigue resistance differentiated higher-ranked U23 and professional climbers, with professionals showing smaller reductions in 1-, 5-, and 20-min efforts under fatigued conditions (Gallo et al. 2022). However, most studies focused on professional or developmental (U23/Junior) cyclists, with recreational cyclists underrepresented; this limits generalizability to broader populations. Sanchez-Jimenez et al. (2023) examined trained/developmental cyclists —with a best 20-min effort of 4.9 ± 0.5 W kg^–1^—and found that performance reductions occurred after an accumulated workload of only 3.5 ± 0.2 kJ kg^–1^, with decrements of 9% (1-min), 5.9% (5-min), and 4.1% (20-min). These findings underscore that durability is closely linked to training status, with higher-level cyclists demonstrating greater fatigue resistance.

### Performance indicator

As previously discussed, decisive moments in professional cycling frequently occur during the final stages of races. This underscores the importance of assessing PO under fatigued conditions, which may serve as a more robust predictor of cycling performance than MMP measured in a rested state (Erp et al. 2021; Leo et al. 2021). In the present study, durability has been primarily evaluated through PO decline during TTs, CP, and power profiling. However, the choice of performance indicator must be carefully considered. While single TTs or power profiles can effectively highlight fatigue-induced PO declines, CP may not adequately capture the impact of fatigue. This limitation stems from CP’s reliance on a mathematical model of maximal efforts (typically 3–12 min), which may not reflect submaximal durability under prolonged fatigue. Short-term anaerobic contributions can also skew CP without indicating sustained performance loss (Poole et al. 2016). Although some studies have reported that CP adequately reflects PO decline (Spragg et al. 2023b; Sanchez-Jimenez et al. 2023), others have demonstrated its inadequacy in this regard (Spragg et al. 2024; Barranco-Gil et al. 2024). For instance, Barranco-Gil et al. (2024) observed a reduction in 2-min PO under fatigued conditions, while 5-min, 12-min efforts and CP remained unaffected. Similarly, Spragg et al. (2024) reported declines in 1-s, 15-s, and 3-min POs following high-intensity efforts compared to a fresh state, whereas 12-min PO remained unchanged. Notably, CP did not differ between fresh and fatigued states in their study, despite clear evidence of fatigue-induced performance reductions. These discrepancies suggest that CP fails to detect fatigue when submaximal capacity, not maximal effort, is compromised. In this sense, the type of fatigue induced—predominantly neural in shorter efforts and metabolic in longer efforts (Voet et al. 2024)—may influence CP’s sensitivity to detecting fatigue. Alternatives like TSS or TRIMP, which integrate intensity and physiological stress, may better capture fatigue effects (Erp et al. 2019a). Practitioners and researchers should thus exercise caution with CP, as it may not fully reflect fatigue’s impact on cycling performance.

### Practical implications

The findings of this study underscore the critical role of cyclist durability in performance, emphasizing that exercise intensity is a primary determinant of the magnitude of PO decline under fatigued conditions. Consequently, implementing targeted training interventions to enhance tolerance to high-intensity efforts may mitigate PO reduction. For example, simulating 5 × 8-min intervals at 105% of CP could mimic Grand Tour stage demands, building resilience to repeated high-intensity efforts (Spragg et al. 2023b). Furthermore, the demands of competitive events can vary significantly across different race types. Monuments like Paris-Roubaix or Tour of Flanders require repeated high-intensity efforts over cobbles or short climbs, unlike flatter stages where steady submaximal power predominates; mountainous Grand Tour stages demand sustained efforts above CP. Analysing the specific demands of races associated with durability, such as the Monuments or mountainous stages in Grand Tours, could provide valuable insights for optimizing training strategies. Specifically, coaches are encouraged to integrate high-intensity, repeated-effort simulations into training programs to better prepare athletes for the physiological and tactical challenges encountered during critical race moments.

### Limitations and future research

One of the main limitations of this study is the heterogeneity of the protocols employed across the included studies, which ranged from 38-min lab rides to 6-h field simulations, complicating direct comparisons and generalizability. Additionally, the lack of a standardized method for quantifying fatigue poses a challenge, as varying metrics (e.g., kJ, TSS, or TRIMP) assessed performance decline differently, potentially affecting accuracy and reliability. The risk of bias assessment further highlights methodological inconsistencies, particularly in group comparability and the selection of non-exposed participants in NOS-assessed studies. Although most studies showed a low overall risk in ROBINS-I and RoB-2, concerns related to confounding factors and missing data were present in a subset of studies. These limitations reinforce the need for future research to adopt standardized fatigue protocols, such as rides at 70% vs. 110% of CP, to enhance consistency, reduce methodological bias, and enable robust meta-analyses.

Additionally, approximately 30% of the included studies were identified through manual screening, which suggests that the initial search strategy may have lacked sensitivity. This limitation is likely due to the omission of variations in terminology typically used in this research area. Indeed, there is a lack of standardised terminology in the literature, leading to the often interchangeable use of fatigue resistance, durability and physiological resilience. Traditionally, fatigue resistance referred to the ability to sustain performance under fatigued conditions. However, recent research –particularly following the introduction of the term durability by Maunder et al. (2021)—has focused more explicitly on quantifying the magnitude of performance decline from a fresh to a fatigued state. The concept of durability has been especially explored in cycling, where the widespread use of power meters allows for accurate measurement of external load and performance loss. More recently, the term physiological resilience has gained traction, reframing the construct around internal load responses, and defined as the ability to resist functional decline following acute and/or chronic stressors (Jones 2024; Jones and Kirby 2025). Given these overlapping yet distinct definitions, future research should aim to clarify and standardize the use of these terms to enable more consistent interpretation and comparison across studies.

Finally, a recent review by Hunter et al. (2025) synthesizes current evidence and highlights that nutritional strategies, particularly carbohydrate intake during prolonged exercise, can significantly modulate durability by influencing the contribution of different metabolic pathways. These findings emphasize the need to account for nutritional variables when assessing performance reductions. Similarly, Peeters et al. (2025) underscore the importance of controlling not only carbohydrate intake during exercise but also in the days prior, an aspect rarely addressed in most studies, thereby compromising the validity and consistency of results. Although this was not a focus of the present review, future studies should consider the role of nutrition when evaluating durability.

## Conclusions

Our findings redefine durability as an intensity-driven phenomenon, shifting the focus from sheer work volume to the ability to withstand high-intensity efforts—an insight that urges a transition from traditional volume-based training to intensity-focused strategies. Rather than merely confirming intensity’s role in PO decline, this study highlights its practical implications: enhancing cyclists’ tolerance to intense efforts could unlock new performance frontiers in competitive cycling. Looking ahead, future studies should validate intensity-adjusted metrics, such as TSS, TRIMP or the novel propose Power Profile Index (PPi) (Mateo-March et al. 2022b), to refine fatigue monitoring and optimize cyclist preparation, ensuring training aligns with the demands of modern racing.

## Data Availability

The data supporting this study’s findings are available from the corresponding author upon reasonable request.
